# ‘Unfit for reform or punishment’: mental disorder and discipline in Liverpool Borough Prison in the late nineteenth century

**DOI:** 10.1080/03071022.2019.1579977

**Published:** 2019-05-10

**Authors:** Catherine Cox, Hilary Marland

**Affiliations:** a University College Dublin; b University of Warwick

**Keywords:** Liverpool Borough Prison, mental disorder, local prisons, punishment, reform, separate system of confinement, penal policy, discipline, prison medical officers

## Abstract

This article examines how Liverpool Borough Prison, opened in 1855 as one of the largest local prisons in England to adopt the separate system, categorized and dealt with mental distress and disorder amongst its prison population in the late nineteenth century. High prison committal rates in Liverpool, alongside high levels of recidivism, especially among female prisoners, led to severe overcrowding and encouraged a harsh disciplinary regime. Exacerbated by the poor physical and mental condition of the prisoners, this produced a challenging environment for maintaining the separate system of confinement and prisoners’ mental well-being. While official figures for the rates of mental disorder in local prisons are not readily available, Liverpool Prison’s diverse and under-exploited archives and official reports indicate that insanity caused prison officials and visiting justices great concern, and many prisoners were declared unfit for the rigours of prison discipline. Our article explores the implications of the ever more punitive, deterrent and physically taxing penal policy implemented in the late nineteenth century on the minds of prisoners. Despite the heavy toll on prisoners’ mental well-being, such cases were often retained by prison medical officers reluctant to acknowledge the failure of the prison to deter, reform and redeem.

In March 1888 Charlotte Oakley (also known as Creswick), a prisoner in Liverpool Borough Prison,^1^1Under the Prison Act, 1865, the term ‘prison’ replaced gaol, house of correction, bridewell, and penitentiary. Though referred to up until 1865 as Liverpool Borough Gaol, for clarity we have used prison throughout the article. The Prison Act, 1865 (28 & 29 Vict c.126). was charged with insubordination. In her mid-20s, Roman Catholic and Irish, Oakley was described by the prison’s visiting justices, warders, doctors and the government inspector, as ‘intemperate’, ‘dangerous to others’ and ‘a prostitute’. During numerous sentences served in Liverpool Prison, Oakley was repeatedly punished for ‘misbehaviour’ and regularly disrupted prison discipline. In October 1888 the government prison inspector questioned Oakley’s mental state and her case was referred to one of the prison doctors, but he did not certify her as insane. With the passage of time, her behaviour spiralled out of control. In August 1892, prison warder, Ellen Bishop, swore that she ‘saw the prisoner break the bolt off the door of her cell’ and strike the prison doctor. As punishment, Oakley was placed in a confinement cell for four days with her hands fastened behind her. In June 1893, she was found to be insane but it was September before she was admitted to neighbouring Rainhill Lunatic Asylum. Now aged 30, on admission to Rainhill she was described as ‘intemperate’, ‘cunning’ and ‘untruthful’, and diagnosed as suffering from mania. She remained in Rainhill for nearly a year – being discharged on 5 September 1894 – but was reported to be back in Liverpool Prison in February 1895. In April 1895, she appeared before the Visiting Committee due to concerns about her mind, was certified by the prison medical officer, Dr Beamish, and readmitted to Rainhill in November 1896. On this occasion, the asylum casebook described her as an ‘alcoholic’ and ‘disorderly prostitute’. No more is heard of Oakley after April 1911, when she was removed from Rainhill to Talgarth Asylum in Wales.^2^2Liverpool Record Office (subsequently LRO), 347 MAG/1/3/3, Proceedings of the Meetings of the Visiting Committee, Liverpool Borough Gaol, April 1878–June 1897, 9 March 1888, 84; 24 October 1888, 96; 29 March 1889, 116; 28 March 1890, 152, 153; 31 Aug. 1892, 224; 11 July 1893, 263; M614 RAI/8/16, Rainhill Asylum Female Casebook, July 1892–March 1894, 178; M614 RAI/8/18, Rainhill Asylum Female Casebook, October 1895–July 1897, 159.

Through a fine-grained study of Liverpool Prison in the second half of the nineteenth century, this article explores the management of inmates like Oakley in local prisons, whose sentences coincided with deteriorating mental health. It asks whether the introduction of separate confinement and subsequent changes to penal policy in the 1860s and 1870s contributed to the incidence of mental disorder among prisoners. New policies introduced in the 1860s and 1870s resulted in punitive, deterrent and physically taxing disciplinary regimes, yet failed to prevent recidivism and a growth in the prison population. We argue that in the context of Liverpool Prison, which in the late nineteenth century was both large and frequently overcrowded, the implementation of these regimes exacerbated the incidence of mental disorder amongst inmates. The article also considers changes in the way mental disorder was dealt with and managed by the prison’s chaplains, doctors, other prison officials and the visiting justices in a city dogged by social problems and high levels of crime.

In recent years, scholarly discourse on the history of penology has moved away from theoretical debates on national penal policy and administration, which preoccupied the field in the 1970s and 1980s, towards the exploration of local prisons, resulting in important studies by Alyson Brown, Seán McConville, Helen Johnston, Barry Godfrey and Lucy Williams.^3^3A. Brown, *Inter-war Penal Policy and Crime in England. The Dartmoor Convict Prison riot, 1932* (Houndmills, 2013), 130–2; A. Brown, *English Society and the Prison. Time, culture and politics in the development of the modern prison, 1850–1920* (Woodbridge, 2003); S. McConville, *A History of English Prison Administration, 1750–1877* (London, 1981); S. McConville, *English Local Prisons 1860–1900. Next only to death* (London & New York, 1995); H. Johnston, *Crime in England, 1815–1880. Experiencing the criminal justice system* (London & New York, 2015); L. Williams, *Wayward Women. Female offending in Victorian England* (Barnsley, 2016); L. Williams and B. Godfrey, ‘Intergenerational offending in Liverpool and the north-west of England, 1850–1914’, *History of the Family*, 20, 2 (2015), 189–203. For women in prisons, see L. Zedner, *Women, Crime, and Custody in Victorian England* (Oxford, 1991) and L. Williams and B. Godfrey, *Criminal Women 1850–1920. Researching the lives of Britain’s female offenders* (Barnsley, 2018). These studies have highlighted the importance of local prisons in terms of the numbers confined within them and the uneven implementation of national penal policy in local contexts. They have also examined expressions of prisoners’ agency such as prison rioting, and prisoners’ identities and interrelationships, with Brown arguing that a ‘microhistory’ approach can reveal much about inmate cultures and the meanings assigned to institutions by various social actors, including prisoners.^4^4Brown, *Inter-War Penal Policy*, *op. cit*., 146. Yet, despite the richness of these works, detailed analysis of the health of prisoners, mental and physical, has not been their primary focus. Important exceptions are the publications of Joe Sim, Peter McRorie Higgins and Anne Hardy, who have assessed the evolving status and role of prison medical officers. Sim has argued that the roots of the Prison Medical Service can be found in the 1774 Gaol Act, while Hardy identifies the Convict Prisons Act of 1850 as pivotal to the emergence of prison medical officers as a distinct professional group.^5^5J. Sim, *Medical Power in Prisons. The Prison Medical Service in England 1774–1989* (Milton Keynes, 1990); A. Hardy, ‘Development of the Prison Medical Service, 1774–1895’ in R. Creese, W.F. Bynum and J. Bearn (eds), *The Health of Prisoners* (Amsterdam and Atlanta, 1995), 59–82. Higgins examines the management and treatment of mental and physical health in English prisons before 1850, contending that surgeons were less concerned with implementing disciplinary regimes than with providing medical care for their charges, including those with mental disorders. This view is shared by Hardy, who concludes that prison medical officers were prepared to circumvent disciplinary instructions considered damaging to prisoners’ health.^6^6P. McRorie Higgins, *Punish or Treat? Medical care in English prisons 1770–1850* (Victoria BC & Oxford, 2007), 236; Hardy, *op. cit*., 77.

Research on the complex relationship between prison regimes and mental disorders – in terms of prisons admitting large numbers of mentally ill people and also as institutions likely to produce or exacerbate mental disease – have largely focused on national and high profile institutions such as Pentonville Model Prison, London, established in 1842 and the Eastern State Penitentary, Philadelphia, opened in 1829. Work by Michael Ignatieff, Ursula Henriques, Margaret DeLacey and William Forsythe has revealed how incidences of mental distress among prisoners were linked to the introduction of the separate system as a disciplinary regime in the Eastern State Penitentiary and at the convict prison at Pentonville, resulting in the toning down of the Pentonville regime in the late 1840s.^7^7U.R.Q. Henriques, ‘The rise and decline of the separate system of prison discipline’, *Past and Present*, 54 (1972), 61–93; M. Ignatieff, *A Just Measure of Pain. The penitentiary in the industrial revolution 1750–1850* (New York, 1978), 11; F. Gray, *Prison Discipline in America* (Boston, 1847); C. Cox and H. Marland, ‘“He must die or go mad in this place”: prisoners, insanity and the Pentonville Model Prison experiment, 1842–1852’, *Bulletin of the History of Medicine*, 92, 1 (2018), 78–109. In contrast to Higgins, Ignatieff concludes that prison surgeons were important actors in maintaining disciplinary regimes and were confident that prisons could produce moral as well as hygienic reform.^8^8Ignatieff, *op. cit*., 59–62. In terms of mental health in local prisons, valuable work by DeLacy and McConville considers whether concerns about the rates of mental disorder among prisoners in local prisons shaped or influenced national policy. While McConville argues that the reformative objective of the separate system had been almost totally eclipsed by the 1850s, William Forsythe contends its local application in county gaols and houses of correction varied considerably, with many larger city gaols, including Liverpool, endeavouring to implement it as fully as possible.^9^9McConville, *English Prison Administration*, *op. cit*., 347; W.J. Forsythe, *The Reform of Prisoners 1830–1900* (Sydney & London, 1987), 93–5. McConville and DeLacy also briefly explore the responses of individual prisons and prison staff and officials to mentally ill prisoners.^10^10McConville, *English Local Prisons*, *op. cit*., 291–2; M. DeLacy, *Prison Reform in Lancashire, 1700–1850. A study in local administration* (Stanford, CA, 1986). McConville focused on the late nineteenth century and identified unsuccessful attempts to halt the admission of cases of insanity into local prisons. Janet Saunders’s work on Warwickshire asylum and prison provides a sustained analysis of the passage of inmates between the two institutions, though her emphasis lies with the certification and disposal of ‘criminal lunatics’ and their treatment at the Warwickshire asylum.^11^11J. Saunders, ‘Institutionalised Offenders: a study of the Victorian institution and its inmates, with special reference to late nineteenth-century Warwickshire’ (Ph.D. thesis, University of Warwick, 1983). In contrast, our article is concerned with the management of insane inmates and the responses of prison medical staff to mentally disordered prisoner/patients within the prison.

From the establishment of the modern prison system, official investigations into penal policy highlighted high rates of mental disorder among prisoners, although most inquiries were preoccupied with the convict prison system, overlooking local prisons.^12^12British Parliamentary Papers (BPP), *Royal Commission on Prisons in Ireland, vol. II*, 1884 (4233–1) XXXVIII.1, Minutes of Evidence, Robert M. Gover, 361. Official figures for the incidence of mental disorder among prisoners in local prisons are not readily available, and, when they were reported, concerned specific groups: the ‘weak-minded or imbecile’ and those with pre-existing disorders on committal. Or they restricted their reporting to the number of prisoners transferred to asylums and the rate of suicide and suicide attempts in prison.^13^13For example, see BPP, *3^rd^ Report of the Commissioners of Prisons*, 1880 (2733) XXXV.1, 28–30. In the year ending 31 March 1895, 389 cases were certified insane in English local prisons, a very low figure and inaccurate reflection of the total number of prisoners suffering some form of mental distress or disorder: J. Baker, ‘Insanity in English local prisons’, *Journal of Mental Science*, 42 (April 1896), 294–302, 297. The Lunacy Commissioners complained that they were unable to accurately establish the number of insane in local prisons, though in their annual report for 1863 they claimed their ‘visitation of gaols’ prompted an increase in removals to asylums.^14^14BPP, *17^th^ Report of the Commissioners in Lunacy* [*RCL*], 1863 (331) XX, 12. Estimates from other contemporary sources indicate that rates in local prisons remained a cause for concern. In his critique of prisons, published in 1894, Reverend William Morrison, chaplain at Wandsworth Prison between 1887 and 1898, claimed insanity rates in local prisons had reached 113 per 10,000 between 1875 and 1877, increasing to 226 per 10,000 in 1890–1892.^15^15W.D. Morrison, ‘Are our prisons a failure?’, *The Fortnightly Review*, 61 (April 1894), 459–69, 468. The administrative records of nineteenth-century local prisons also comment regularly on high rates of mental distress among male and female prisoners, despite efforts to play this down and to attribute it to prisoners’ enduring attempts to feign insanity.

Liverpool Prison, which opened in 1855, was large, yet frequently overfull; many prisoners were on short-term sentences and there were high rates of recidivism. It also had an unusually high proportion of female prisoners. While most prisoners were serving short sentences and not undergoing sustained periods in separation, the Liverpool justices and prison governors remained committed to upholding the separate system in the 1850s and 1860s when it retained some of its reformative elements and their commitment, while often thwarted, went beyond ‘lip service’.^16^16McConville, *English Prison Administration*, *op. cit*., 354. This article argues that conditions at the prison challenged this aim and some prison staff came to support the short, sharp, shock of a harsher disciplinary regime following the introduction of revised prison rules in the 1860s and the nationalization of the prison system after 1877.

As our analysis of the minutes of the meetings of visiting justices and annual reports of Liverpool’s governors, medical officers and chaplains highlight, managing mentally disordered prisoners became a significant part of the prison staff’s workload and a financial drain. Local authorities were obliged to meet the cost of maintaining prisoners in asylums, a requirement they were unhappy with. Liverpool Prison officials carefully monitored the mounting expense of paying for these prisoners – categorized as ‘private patients’ in asylum records, compounding the difficulties faced by the Lunacy Commissioners in tracking their numbers. Many prisoners suffering from mental disorders remained in local prisons as officials were unable to decide – or agree – on a prisoner’s state of mind. As with Creswick Oakley, they were slow to make transfers, because of financial considerations but also as it indicated the failure of the institution and its staff to manage its prisoners. They were also wary of removing feigners or malingerers whose ‘insanity’ was doubted; such prisoners, it was argued, were eager to secure removal to asylums where they benefitted from the ameliorated conditions or were able to escape from custody.

Even so, some prisoners were eventually diverted into expensive criminal or public asylums, often following protracted prison careers characterized by re-committals to prison and repeated breaches of prison discipline and punishments. Alienists and advocates of specialized asylum treatment had long insisted that prisons, as well as workhouses, were inappropriate places for the insane, criminal and non-criminal, criticizing prison doctors for their poor understanding of mental illness.^17^17F. Driver, *Power and Pauperism. The workhouse system, 1834–1884* (Cambridge, 1993), 108. Yet, asylum superintendents and the Lunacy Commissioners were resistant to the reception of prisoners into their, often overstretched, institutions, regarding them as tainted by their criminality, troublesome, disruptive and likely to contribute to high mortality rates.

Our article draws on the diverse and under-exploited archives of Liverpool Prison, including annual reports and minute books, as well as official accounts and newspaper reports. In addition, we tracked the transfer of individuals from Liverpool Prison to the neighbouring lunatic asylums of Rainhill, Prestwich and Lancaster through a close reading of the minutes of the prison’s visiting committee and annual reports. The extant records for Liverpool Prison do not facilitate systematic, large-scale record linkage of all the transfers; very few casebooks for Prestwich and Lancaster asylums have survived and there are gaps in the runs of Rainhill Asylum’s male and female casebooks. However, when a prisoner was named in the prison minute books and annual reports and details of the asylum noted, we were able to locate the casebook entries for these individuals. We also consulted Rainhill’s surviving casebooks for instances where prisoners were identified as being transferred from Liverpool Prison. The records for Rainhill Asylum are the most complete of the three Lancashire asylums, and the majority of cases discussed in this article were transferred there. Through this method we have been able to map the status of approximately 40 prisoners/patients or ‘lunatic criminals’ moved from prison to asylum, and, in some cases, their return to prison between 1870 and 1897.

## The early years of the separate system of confinement

The introduction of the separate system of confinement to local and convict prisons in England followed decades of deliberation about the relationship between punishment and reformation. From the late eighteenth and into the early nineteenth century there were extensive debates on the benefits of the regime over the rival silent system, which had been introduced in the Auburn and Sing Sing Penitentiaries in New York State.^18^18For more on the silent system, see R. McGowen, ‘The well-ordered prison: England, 1780–1865’ in N. Morris and D.J. Rothman (eds), *The Oxford History of the Prison. The practice of punishment in western society* (Oxford, 1998), 71–99, 90; D.J. Rothman, ‘Perfecting the prison: United States, 1789–1865’ in Morris and Rothman, *op. cit.*, 100–16, 108. The Gloucestershire magistrate, Sir George Onesiphorus Paul, for example, introduced a regime of complete separation similar to separate confinement in his county gaol as early as 1791.^19^19Forsythe, *The Reform of Prisoners*, *op. cit*., 15–29; DeLacy, *op. cit*., 205–6; N. Herbert, ‘Paul, Sir George Onesiphorus, (1746–1820)’, *Oxford Dictionary of National Biography*, https://doi.org/10.1093/ref:odnb/21597 (accessed 23 April 2018). While there was fierce opposition to the separate system from prison governors such as George Laval Chesterton, Governor of Cold Bath Fields Prison in Middlesex from 1829 to 1854, by the 1830s evangelical and spiritual reformers, keen and influential advocates of the system, had become convinced of its potential to reform. Its use had been enshrined in the 1779 Penitentiary Act which allowed for its implementation at Millbank Penitentiary when it was first occupied in 1816, while the 1839 Prison Act favoured and regularized separation in local gaols, although the legislation did not compel local officials to implement it.^20^20Ignatieff, *op. cit*., 171; McConville, *English Prison Administration*, *op. cit*., 254.

Though introduced to several local prisons, including Preston and Kirkdale Gaols in Lancashire, by the early nineteenth century it was at Pentonville convict prison where the fullest and most rigorous form of the separate system was implemented.^21^21Forsythe, *The Reform of Prisoners*, *op. cit*., 29–34; W.J. Forsythe, ‘The beginnings of the separate system of imprisonment 1835–1840’, *Social Policy and Administration*, 13, 2 (1979), 105–10. There, prisoners were forbidden from communicating with each other, and spent almost 23 hours of each day in their solitary cells, where they worked, ate and slept.^22^22The National Archives (subsequently TNA), PCOM Second Report, 18–20, Appendix B, No. 3 ‘Routine of a Day’, 190; Ignatieff, *op. cit*., 3–9. In contrast to the kinds of prisoners that Liverpool would subject to separate confinement, initially Pentonville’s convicts were hand-picked – aged between 18 and 35, and in robust health, fit to withstand what even Pentonville’s supporters would deem a rigorous form of discipline. Critics of Pentonville, including eminent psychiatrist Dr Forbes Winslow, novelist Charles Dickens, and *The Times* newspaper, were strident in their opposition to the system, particularly as the Pentonville regime became associated with high rates of mental distress among its prisoners.^23^23F. Winslow, ‘Medical Society of London: prison discipline’, *Lancet* (22 March 1851), 357–60, 359; ‘American notes’, *The Times*, 25 Oct. 1842, 3. In acknowledgement of this, in 1847, its rigour was toned down, and the length of separation gradually reduced from 18 to nine months by 1853.^24^24Henriques, *op. cit*., 86.

Despite the public failure of the Pentonville ‘experiment’, and the revelations about the damage inflicted on prisoners’ minds, support for modified forms of the separate system of confinement, which focused less on reforming prisoners, remained entrenched in penal policy and would shape and dominate prison disciplines and philosophies across England into the twentieth century.^25^25M. Ogborn, ‘Discipline, government and law: separate confinement in the prisons of England and Wales, 1830–1877’, *Transactions of the Institute of British Geographers*, 20 (1995), 295–311; Cox and Marland, *op. cit.* Responding to events at Pentonville, from the 1850s a revised version of the separate system was introduced to the government convict system. In the new generation of purpose-built ‘modern’ local prisons, including Liverpool Prison, as well older local prisons, implementation of the separate system was further endorsed and strengthened. The 1850 Select Committee on Prison Discipline, chaired by Home Secretary Sir George Grey, supported the introduction of ‘entire separation’ throughout English local gaols with some modifications introduced to prisoner’s routine during labour and religious worship, though they were still prohibited from breaking the rule of silence.^26^26*Report from the Select Committee on Prison Discipline together with the Proceedings of the Committee, Minutes of Evidence, Appendix and Index* [Grey’s Committee], 1850, 126. In terms of practical implementation in local gaols and houses of correction, there was significant variation. Nonetheless some local gaols, Liverpool amongst them, were committed to it as a disciplinary and reformative regime.

Local gaols housed the vast majority of English prisoners and according to influential penal reformer and politician Lord Carnarvon were of greater importance to national penal policy than convict prisons.^27^27McConville, *English Local Prisons*, *op. cit*., 98. They differed from convict prisons with their mixed populations of inmates convicted of minor misdemeanours, serving shorter sentences or awaiting trial as well as a small but, in the case of Liverpool, significant cohort of convicted government prisoners who were held in the prison while awaiting transfer to the convict system. With a rapid turnover of large numbers of prisoners, local prisons were more likely to be subject to overcrowding and poor conditions, and commitment to imposing separate confinement was patchy. In some, only a portion of the prison made provision for separation, or it broke down as the prison became overcrowded.^28^28BPP, *Report from the Select Committee of the House of Lords on the Present State of Discipline in Gaols and Houses of Correction* [Carnarvon Committee], 1863 (499) IX, iii–vi. While there was variation between local prisons with regard to diet, labour and punishment, they were often typified by harsh conditions and disciplinary regimes that imperilled the physical and mental health of their prisoners, and Liverpool was no exception to this.

## Liverpool Prison and the doctrine of separate confinement

When it opened in 1855 Liverpool Prison, with its 1,000 cells, functioned as a local prison and convict repository and was, according to Governor William Jameson, ‘the largest in England, if not in Europe on the separate system’, as well as ‘the only prison in the world where the females exceed the males’.^29^29Quotes from LRO, 347 MAG/1/2/1, Minutes of the Quarterly and Annual Meetings of the Visiting Justice of the Borough Gaol and House of Correction, 1852–1864, 6 Feb. 1857, 76. See also *Liverpool Mercury*, 7 Sep. 1857; J. Belchem, *Irish Catholic and Scouse. The history of the Liverpool-Irish, 1800–1940* (Liverpool, 2007), 82; Brown, *English Society and the Prison*, op. cit., 47. From the start the justices at Liverpool Prison intended to implement the separate system of confinement although, as explained below, they realized conditions in Liverpool Prison would make this extremely difficult. Yet, it remained their aim and in 1856 a return submitted by local prison officials detailing the implementation of separate confinement across local and convict prisons found the regime was operating fully in Liverpool, though it also noted that some prisoners were permitted to work in association following a probationary term in separation.^30^30BPP, *Prisons (Separate Confinement)*, 1856 (163), XLIX, 3. Designed for the separate system and cellular isolation Liverpool Prison was a ‘parallelogram’ with cells, four tiers in height, running along the corridors with the male and female sections divided internally. The footfall and turnover of prisoners was remarkable; in 1857 it was estimated that about ‘10,000 persons graduate[d]’ through the building with an average number of ‘upwards of 30 [prisoners] a day’ being received and discharged.^31^31*Liverpool Mercury*, 14 Sep. 1857, 4. Confined to their cells, prisoners started work at 6 am, retiring at 8 pm and were under constant supervision:

in the door of the cell … there is a pierced eye-hole by which the turnkeys can look in upon the prisoner at any time, without being observed by him .… The [prison] officers are to be seen quietly walking about, and although there is a population of above 1200 persons within a very narrow compass everything is as silent as the tomb, the light sombre, the whole effect saddening and impressive.^32^32*ibid.*, 7 Sep. 1857, 5.

While Liverpool Prison was the largest prison in Lancashire to adopt the regime, it was not the first. Under the influence of John Clay, a national authority on crime and punishment, ardent supporter of the separate system and prison chaplain at Preston Gaol from 1823, most of Lancashire’s prisons had adopted substantial elements of separation.^33^33B. Forsythe, ‘Clay, John (1796–1858)’ in *Oxford Dictionary of National Biography*; online edn, Jan. 2008, http://www.oxforddnb.com/view/article/5561 (accessed 15 Dec. 2016). Regarding crime as a ‘moral individual failing’ requiring the aids of religion and education to bring about reformation, Clay persuaded the county’s newly appointed visiting justices to introduce separation at Preston and Kirkdale Prisons after 1846.^34^34*ibid*.; DeLacy, *op. cit*., 220. Despite enthusiastic support from other chaplains, including Richard Appleton, chaplain at Kirkdale Prison, it was not always possible to fully implement the system in older prisons not designed for separate confinement. In April 1856 Inspector of Prisons, Herbert P. Voules, reported that there were 142 cells at Preston and 199 at Kirkdale which were unfit for separation and prisoners occupying these cells were ‘exposed to the corrupting influence of the associated system’.^35^35BPP, *19^th^ Report of the Inspectors of Prisons of Great Britain. Northern and Eastern Districts* [*RIPGB*], 1856 (2102), XXXIII, 5. It was also reported that the silent system, prohibiting communication among prisoners at all stages of their sentence, including associated work, and enforced through punishments, was in operation at Preston.^36^36BPP, *Prisons (Separate Confinement*), 3; Forsythe, *The Reform of Prisoners, op. cit*., 30–5. Given the prison’s size and the type of prisoner incarcerated there, Liverpool Prison’s chaplain, Thomas Carter, and the visiting justices were concerned about the feasibility of implementing separate confinement.^37^37LRO, 347 MAG/1/2/1, Minutes VJ, 23 July 1855, 35. It is, however, unclear whether Carter or other prison officials at Liverpool were aware of the wider debates on the adverse impact of separate confinement on prisoners’ minds or if there were conversations with Appleton and Clay at Kirkdale and Preston. Yet immediately after Liverpool Prison opened, the difficulties of fully implementing separation became apparent.

Separate confinement as designed for convict prisoners and implemented at Pentonville was intended for carefully selected prisoners who were serving long sentences, a point emphasized by Reverend John Burt, Deputy Chaplain at Pentonville from 1843. In his 1852 defence of the system, Burt insisted that shortening the time prisoners spent in separation and the relaxation of the selection process had resulted in an increase in the numbers suffering from mania and delusions.^38^38J.T. Burt, *Results of the System of Separate Confinement as Administered at the Pentonville Prison* (London, 1852), 96–7. In Liverpool the situation was very different. The fact that most Liverpool prisoners were serving short sentences for minor offences, felonies or misdemeanours, or were in prison awaiting trial, meant that, ostensibly, they were likely to be confined in separation for relatively short periods often as little as one month, and were less likely to benefit from its reformatory effects. In 1856, over 73% of committals were for one month or less and a further 10% between one and two months.^39^39BPP, *22^nd^ RIPGB*, 1857–58 (2373), XXIX, 19. Prisoners were often in very poor physical condition and included very young prisoners as well as ‘old offenders’. Alongside short-term prisoners, government convicts, committed for penal servitude, were temporarily detained at Liverpool until they could be removed to a convict prison, although Liverpool, unlike other local gaols, did not rent cells to the government for convicts.^40^40McConville, *English Prison Administration, op. cit*., 429. At times government convicts accumulated – in September 1857 there were 102 awaiting transfer – contributing to the prison’s consistently overcrowded state.

A particular problem on the female wing, overcrowding prompted modifications to the separate system. In October 1855, a month after the prison opened, the visiting justices observed there were only 407 cells for between 416 and 429 female prisoners. In October, the governor permitted women to sleep in association in cells or in the prison hospital, and in May 1857 straw beds were supplied for ‘doubling-up’ in cells.^41^41LRO, 347 MAG/1/2/1, Minutes VJ, 27 Oct. 1855, 50; 20 May 1857, 82. The large number of recommittals, especially among women, exacerbated problems. In 1856 the justices and inspector Voules noted that rates of recommittal had increased by 2.7% and a large proportion of these, 34.1%, were ‘old offenders’ with four or more previous convictions.^42^42BPP, *22^nd^ RIPGB*, 21. Recommittal rates were higher for women, up by 5%.^43^43LRO, 347 MAG/1/2/1, Minutes VJ, 7 Feb. 1856, 53. These prisoners were recommitted for minor offences, such as vagrancy, prostitution, drunkenness and indecent conduct, and served short sentences.^44^44BPP, *22^nd^ RIPGB*, 21. Chaplain Carter, commenting on ‘41 women committed on one day’, observed that their ‘united previous convictions amounted to 467’.^45^45*ibid.*, 23. The pressure on the female wing was temporarily eased in early 1859 after the removal of 56 government convicts – 17 male and 39 female – but by May 1859 the separate system had again collapsed.^46^46LRO, 347 MAG/1/2/1, Minutes VJ, 22 May 1859, 129. In 1863 overcrowding throughout the prison meant that single occupancy cells were used to accommodate three prisoners on the male wing and two on the female. On this occasion Voules suggested two dormitories be ‘fitted up’ to accommodate the excess numbers at night.^47^47BPP, *29^th^ RIPGB*, 1864 (3326), XXVI, 20. Yet, despite these pressures, the full implementation of separate confinement remained the ambition of the visiting justices and the governor.^48^48LRO, 347 MAG/1/2/1, Minutes VJ, 14 Jan. 1859, Governor’s Report, 118.

Liverpool Prison, serving a major port and expanding commercial city, faced huge pressures in term of high rates of crime, exacerbated by poverty and hardship, drinking and prostitution. The city attracted large numbers of mainly Irish migrants, seeking often low-skilled and casual employment in the city’s docklands.^49^49F. Neal, *Sectarian Violence. The Liverpool experience, 1819–1914: an aspect of Anglo-Irish history* (Manchester, 1988); M. Macilwee, *The Liverpool Underworld. Crime in the city, 1750–1900* (Liverpool, 2011). During the Irish Famine (1845–1851), huge numbers migrated to Liverpool; between 1841 and 1851 the Irish-born population of the city rose from 17.3% of the total population to 22.3%. In popular perception and vitriolic local press coverage, the Irish were associated with bringing disease, unrest and crime into the city, and crime rates amongst the Irish reflected these anxieties. Large numbers of impoverished Famine migrants ended up in Lancashire prisons, often under vagrancy legislation.^50^50*Manchester Times*, 15 Jan. 1851, 4; Neal, *op. cit*., 97. The rate of emigration eased in the post-Famine years, but still remained high, 12.6% of the population in 1891.^51^51Neal, *op. cit*., 7–9. Irish-born migrants and the ‘culturally Irish’ – Liverpool-born Irish – were strongly over-represented in crime statistics. In 1871 they accounted for 33.4% of arrests in the city, mostly for assaults and vagrancy, although as John Walton et al. have argued, the association with criminality eased at the end of the nineteenth century and was accompanied by a decline in anti-Irish rhetoric in the city’s press.^52^52W.J. Lowe, *The Irish in Mid-Victorian Lancashire. The shaping of a working-class community* (Bern & New York, 1989), 38–9; J.K. Walton, M. Blinkhorn, C. Pooley, D. Tidswell and M.J. Winstanley, ‘Crime, migration and social change in North-West England and the Basque Country, 1870–1930’, *British Journal of Criminology*, 39, 1 (1999), 90–112, 109. Nonetheless, between 1864 and 1879, 65% of those committed to Liverpool Prison were Catholic and 9 out of 10 of these were Irish-born or the children of Irish-born parents.

The Irish preponderance was accentuated among women. In his report for 1868, the Roman Catholic prison minister and temperance advocate, James Nugent, noted that 69% of committals were comprised of female, Catholic prisoners in comparison to 57% among men and most were illiterate or near illiterate.^53^53LRO, 347 JUS/4/1/2, Minutes of Justices Sessions Gaol and House of Correction (MJ), October 1864–1870, Prison Minister’s Report (1868), 16, 17; Nugent was appointed under the 1863 Prison Ministers Act, see McConville, *English Local Prisons*, *op. cit*., 279. For Nugent, see Belchem, *op. cit*., 81–7; D. Beckingham, *The Licensed City. Regulating drink in Liverpool, 1830–1920* (Liverpool, 2017), 79–80. Large numbers were charged with prostitution – in the first nine months of 1864, 1,526 prostitutes were taken into the prison – or of being drunk and disorderly. Some women had 30, 40, 50 or even 60 prior convictions, with short-term sentences.^54^54LRO, 347 JUS/4/1/2, MJ, Prison Minister’s Report (1864), 20–1. In 1871 one woman who had been in prison six times in the last quarter year, ‘attained her 105^th^ commitment’.^55^55LRO, 347 MAG/1/2/2, Proceedings of the meetings of the Liverpool Justices of the Peace, Minutes 1870–1878, Prison Minister’s Report, 27 April 1871, 44. The high rates of committals among women – Irish and non-Irish – was linked to limited employment opportunities in Liverpool and their dependence on casual and poorly paid work.^56^56J.K. Walton, *Lancashire. A social history, 1558–1939* (Manchester, 1987), 287–8. Among the women recorded as prostitutes, Nugent estimated that approximately 60% were Catholic.^57^57LRO, 347 JUS/4/1/2, MJ, Prison Minister’s Report (1868), 20–1. For a discussion of prostitution and intemperance in Liverpool, see Beckingham, *op. cit*., chs 5 and 6. In the 1850s, there were reportedly ‘695 brothels, 81 houses of accommodation and 102 houses where prostitutes lodge’ in Liverpool, with over 2,000 women and girls ‘known as professed prostitutes’.^58^58BPP, *22^nd^ RIPGB*, 23. Reflecting on the situation, Governor Jackson observed in 1859:

No system of prison discipline will have the greatly desired effect of either deterring or reforming these immoral and depraved women, so as to prevent them returning to their dissolute and intemperate habits, while there are so many receptacles ready for them, and so many inducements and facilities afforded to them in Liverpool.^59^59BPP, *25^th^ RIPGB*, 1860 (2645), XXXV, 31.

Hoping to reduce the high incidence of recommittals among women, especially those charged with prostitution (many of whom were said to be recruited in prison), Chaplain Carter, and his successors advocated for additional support, including the establishment of the Discharged Prisoner’s Aid Society for women, to avoid a return to the corrupting and contaminating environment of Liverpool city.^60^60BPP, *22^nd^ RIPGB*, 23.

## Liverpool Prison and mental disorders, 1855–1865

Incidences of mental disorder among prisoners at Liverpool Prison began to be disclosed shortly after it opened. In one of his first visits to the prison in 1856, Inspector Voules reported on a 12-year-old boy who had committed suicide after three weeks’ confinement with just five weeks remaining of his sentence.^61^61*ibid*., 22; See McConville, *English Local Prisons*, op. cit., 295–8, for a discussion of suicides in local prisons in the 1880s. One male and one female prisoner were moved to local lunatic asylums. The male prisoner was described as being of ‘weak intellect and subject to fits of violence before imprisonment’. He had displayed signs of insanity three weeks after committal and was transferred to Rainhill Asylum one month later. The unnamed woman had been committed to Liverpool Prison on 33 occasions. Symptoms of insanity were noted on her arrival and she was moved to Lancaster Asylum within three weeks.^62^62BPP, *22nd RIPGB*, 22.

The arrival of insane prisoners at lunatic asylums was not always welcomed, despite alienists’ claims of professional expertise and their insistence that prisons and workhouses were unsuited for the insane. In 1854, when advocating for state criminal asylums – Broadmoor Criminal Lunatic Asylum was opened in 1863 – the Lunacy Commissioners commented that mentally ill prisoners were ‘morally tainted with crime’ and unfit for association with other asylum patients.^63^63BPP, *8^th^ RCL*, 1854 (339), XXIX, 47. For Broadmoor, see J. Shepherd, ‘“I am very glad and cheered when I hear the flute”: the treatment of criminal lunatics in late-Victorian Broadmoor’, *Medical History*, 60, 4 (2016), 473–91; M. Stevens, *Broadmoor Revealed. Victorian crime and the lunatic asylum* (Barnsley, 2011). The Committee of Visitors at Lancaster Asylum agreed, complaining of ‘the inconvenience and evils of their [criminal lunatics] confinement and association with the ordinary inmates of our Asylums’.^64^64Wellcome Library (subsequently WL), Reports of the County Lunatic Asylums at Lancaster, Prestwich and Rainhill, 1854, Report of the Committee of Visitors, Lancaster, 11. For asylum employees, such patients were troublesome, requiring extra staffing due to the risk of escape and because of the type of illnesses they brought into the institution,^65^65BPP, *16^th^ RCL*, 1862 (417), XXIII, 133. notably general paralysis of the insane; they required additional nursing and had high mortality rates.^66^66WL, Reports of the County Lunatic Asylums at Lancaster, Prestwich, and Rainhill, 1855, Report of Superintendent, Rainhill Asylum, 88. In March 1853 the medical superintendent at Prestwich Asylum reported that of the 399 patients there at the start of the year, 99 were subject to epilepsy and general paralysis.^67^67WL, Reports of the County of Lancaster Lunatic Asylum at Prestwich, 1852, Report of Superintendent, 14. ‘Lunatic’ prisoners accumulated in asylums, were unlikely to recover and were often transferred to other asylums rather than being discharged. In 1858, nine criminal cases were admitted to Rainhill; 10, who had been admitted the previous year, remained. By 1862 Lancaster Asylum held 24 criminal patients though the asylum superintendent, John Broadhurst, insisted only four or five of these were suitable for removal to the criminal lunatic asylum.^68^68BPP, *16^th^ RCL*, 131.

Responding to incidences of mental breakdown, Liverpool Prison’s officers conceded that separate confinement might exacerbate disorders among those with a predisposition to insanity or who were weak-minded. In his 1856 report, Governor Anderson observed that

in the number of persons who are from time to time committed to this prison, there are some who are predisposed to insanity, or whose minds are weak, or who are subject to delusions or other mental affections, and it is very probable that separate confinement may have a tendency to cause such maladies to be more fully developed. Whenever such cases occur the prisoner is closely watched, and the surgeon’s opinion immediately taken, and when necessary the prisoner is removed from the cell to work in association during the day, and if requisite other prisoners sleep in the same room during the night.^69^69BPP, *22^nd^ RIPGB*, 22.

They observed and commented on the strain prisoners endured while undergoing separation even for a relatively short time, in extreme cases prompting prisoners to self-harm or feign suicide and mental disorder. During his 1856 visit, Voules reported on a male prisoner who threw himself from an upper gallery and a woman who tried to hang herself in her cell. Both survived and were discharged at the end of their sentences. Voules insisted that ‘six other prisoners’ had ‘feigned attempts to hang themselves, with a view to procure removal from separate confinement’.^70^70ibid. Investigations into attempts at self-harm and suicide were common and often dismissed as shamming and prisoners punished or placed under the observation of other prisoners or the prison doctor. Governor Anderson, however, also claimed that some prisoners were grateful to be separated from ‘old associates’ and appreciated the ‘benefits’ of separate confinement. He recalled the case of a male prisoner previously confined in the old Liverpool Prison, who allegedly told the governor ‘with much earnestness’ that ‘if I had been sent into a place like this at first I should never have continued a thief; it was seeing other bad prisoners inside, and meeting them again outside, that has brought me to this’.^71^71ibid. Other prisoners, however, clearly dreaded the prospect of imprisonment; in 1865, two committed suicide immediately on receiving their sentences.^72^72LRO, 347 JUS/4/1/2, MJ, 3 Nov. 1865, Surgeon’s Report, 44–5.

The presence of the mentally ill in prison prompted tensions among staff at a time when prison doctors sought to distinguish themselves from their generalist predecessors and to enhance their status.^73^73Hardy, *op. cit*., 60. In the early years of the separate system chaplains, including those at nearby Preston and Kirkdale, were self-declared ‘experts’ in matters relating to prisoners’ minds. In Liverpool Prison the burden of investigating cases of mental disorder, organizing removals to other institutions and looking out for signs of feigning, increasingly fell to the hard-pressed doctors as well as the chaplains. By 1855 prison rules, which were based on those laid down by the 1823 Gaol Act, enhanced the role of the doctor and charged him with visiting every prisoner twice a week, while prisoners in solitary confinement or close confinement were to be visited daily.^74^74*ibid*.; DeLacy, *op. cit.* If the doctor believed ‘the mind or body of a prisoner is likely to be injuriously affected by the discipline or treatment’, he was to report the case. Through this close observation, high profile prison doctors, including Dr John Campbell of Woking Invalid Prison, Dr William Guy, medical superintendent of Millbank Prison from 1859 to 1869, and Dr Robert Gover, who had worked in the convict service from 1857 and was appointed medical inspector of local prisons in 1878, claimed expertise in understanding mental illness in the prison context. They adopted the term ‘lunatic criminals’ to distinguish the class of prisoner patient they were dealing with, prisoners found to be insane once they had entered prison. They also asserted a particular knowledge of feigning or malingering, framing it as a form of deviance and disorder of the mind and morals to which criminals were likely to be prone.

Meanwhile, the influence of the chaplain was declining but still significant. His role was circumscribed under the 1855 prison rules though he continued to be accredited with particular expertise in matters related to education and prisoners’ minds. In preparation for the introduction of separate system, for example, Liverpool’s chaplain was tasked with determining the number of teachers to be employed, seeking advice from chaplains at Birmingham, Preston, Manchester, Leeds, Wakefield, Stafford and Wandsworth Prisons.^75^75LRO, 347 MAG/1/2/1, Minutes VJ, 23 July 1855, 37–8. The 1855 rules specified that the doctor was to alert the chaplain to prisoners requiring ‘special care’, and to ‘pay attention to the state of mind of prisoners’.^76^76LRO, 347 JUS/4/2/1, Rules and Regulations for the Government of the Liverpool Borough Gaol and House of Correction at Walton-on-the-Hill, near Liverpool (1855), 31–6, 31, 34, 36. At Liverpool, these regulations led to an increase in the workload of chaplains and doctors and become a source of conflict.

By the early 1860s Liverpool Prison’s surgeon Dr Francis Archer was closely monitoring prisoners for signs of mental disorder. In 1863 he relaxed the ‘severity of the discipline’ for six prisoners who ‘exhibited, more or less, tendency to aberration of the mind’, and one woman was removed to an asylum.^77^77BPP, *28^th^ RIPGB*, 1863 (3234), XXIII, 61. In his 1864 report, Archer commented on seven prisoners he had removed from the separate system into association as they ‘exhibited symptoms of insanity’.^78^78LRO, 347 JUS/4/1/2, MJ, Surgeon’s Report (1864), 22. While separation had been ‘instrumental in developing the disease’ in two of the cases, he concluded that among the remaining five, the ‘affection might have exhibited itself’ had they not been in prison. Three of the prisoners were removed to Rainhill Asylum; Archer organized for the other two to be sent to the workhouse on the expiration of their sentences.^79^79BPP, *29^th^ RIPGB*, 1864 (3326), XXVI, 21; LRO, 347 JUS/4/1/2, MJ, Surgeon’s Report (1864), 22. Removals to local workhouses were not unusual. In his report, Archer commented on five such instances, including a ‘remarkable case of self mutilation which occurred in a prisoner who had previously been in an asylum, but who up to the time he committed the act, or afterwards, did not betray any symptoms of unsound mind’.^80^80LRO, 347 JUS/4/1/2, MJ, Surgeon’s Report (1864), 22. Removal to workhouses was also used in the management of ‘sick, infirm and destitute’ prisoners on their discharge, and to contain outbreaks of epidemic diseases.^81^81*ibid*., 3 Nov. 1865, Surgeon’s Report, 44–5. See McConville, *English Local Prisons*, *op. cit*., 289 for examples of removals from local prisons to workhouses to isolate infectious prisoners. By 1868, the prison governor had established a formal arrangement with the guardians of the West Derby Union, who agreed to accept prisoners into the workhouse though there was resistance to the reception of ‘dying’ prisoners.^82^82*ibid*., 29 Oct. 1868, Governor’s Report, 12; Letter from Clerk of Union, 27 Aug. 1868, 170–1; BPP, *25^th^ RIPGB*, 1860 (2645), XXXV, 32.

Prisoners removed to Rainhill Asylum had diverse histories of convictions and imprisonment and varied manifestations of mental disorder. William Smith, a 35-year-old widower and clerk, was admitted to Rainhill from Liverpool Prison in February 1860, where he had been awaiting trial for ‘walking away with another man’s hat from a church’. Smith had been in prison in similar circumstances in 1858 when he had been committed for stealing a book and coffee pot and showed symptoms of insanity while on remand. In a report to the Home Office, governor William Jameson commented that Smith had been insane for two months and laboured under ‘the delusion that he is a person of station .… He imagines he is about to be appointed Assistant Chaplain to the prison’, but was also ‘submissive and inoffensive’ with ‘cleanly’ habits.^83^83TNA, MH51/152, Liverpool Borough Gaol, Lancashire, 11 March 1858. While Jameson’s report does not specify the outcome of Smith’s case, he appears to have been committed to Rainhill but was subsequently discharged. Within two months of his release, Smith paid a visit to the asylum and was described in the asylum case notes as being ‘undeniably insane’. He was not readmitted to Rainhill but reappeared at Liverpool Prison in 1860 and was again removed to Rainhill in February, when his intellect was described as being ‘weaker’, the admitting doctor fearing there were ‘more decided symptoms of the development of organic disease’. He never returned to prison but was removed to West and East Riding Asylum at York on 23 February 1860.^84^84LRO, M614 RAI /11/2, Rainhill Asylum Male Casebook, Feb. 1857–May 1861, 113.

## Liverpool and prison policy 1860s–1870s

Throughout the mid-1860s and 1870s, there was wide-felt discontent concerning the effectiveness of England’s prison system, fuelled by claims that it did not deter repeat offenders and that crime was increasing and becoming more brutal. By the 1860s, the emphasis of English prison policy had shifted from rehabilitation and reform towards a more punitive and deterrent system, which presented prisoners as a dangerous class, incapable of reform. Local prisons were criticized for failing to impose rigorous and uniform systems of discipline, for overfeeding prisoners and being lax in the oversight of ticket-of-leave prisoners, though it was acknowledged that in local prisons sentences were too short for the full application of the separate system.^85^85Forsythe, *The Reform of Prisoners*, *op. cit*., 146–9. This growing dissatisfaction prompted Lord Carnarvon’s 1863 House of Lords Select Committee on Prison Discipline and culminated in two Prison Acts in 1864 and 1865.^86^86McConville, *English Local Prisons*, *op. cit*., 97–148. Under new rules, separation remained intrinsic to the English prison system, but assumed an expressly punitive function. Prisoners were to be isolated at night and put to work at hard labour by day. To incentivize good behaviour, ‘the possibility of promotion to a less arduous stage by obedience and docility’ was introduced.^87^87Forsythe, *The Reform of Prisoners*, *op. cit*., 160.

These changes continued with the appointment of Edmund Du Cane as Chairman of the Prison Commission and of the Directors of Convict Prisons from 1869, and the centralization of the prison system after the 1877 Prison Act. Du Cane criticized the spiritual reformists of the 1840s, dismissing their philosophies as naïve and absurd. Convinced that most prisoners were incapable of reform, he supported cellular isolation with hard labour and his era came to be characterized by the enforcement of a uniform national system of discipline opposed to variation and autonomy in local prisons.^88^88B. Forsythe, ‘Du Cane, Sir Edmund Frederick (1830–1903)’ in *Oxford DNB*; online edn, Jan. 2008, http://www.oxforddnb.com/view/article/32910 (accessed 19 Feb. 2017); Forsythe, *The Reform of Prisoners*, *op. cit*., 196–7. This left little room for individualization – the one-on-one cell visitations for reflection to ‘treat’ and reform prisoners – at the core of separate system in the 1840s. The 1877 Prisons Act enforced a ‘reward’ or marks system in local prisons, whereby prisoners were promoted through a system of graduated classes, differentiated by the severity of labour, entitlement to exercise, diet and sleeping conditions, and on release they were paid small sums of money. Most prisoners in local prisons, such as Liverpool, were unlikely to benefit from the graduated marks system as they were on short sentences, and instead were subjected to harsh disciplinary regimes aimed at recidivist prisoners in an effort to deter rather than reform them.

These approaches to penal policy coincided with evolving psychiatric theories on the nature and cause of criminality. While English alienists rejected continental theories of the ‘born criminal’, put forward by Cesare Lombroso and his associates, there was growing support for the existence of a habitual and irredeemable criminal ‘type’.^89^89N. Davie, ‘Criminal man revisited? Continuity and change in British criminology, c.1865–1914’, *Journal of Victorian Culture*, 81, 1 (2003), 1–32; S. Watson, ‘Malingerers, the weakminded criminal and the “moral imbecile”: how the English prison medical officer became an expert in mental deficiency, 1880–1930’ in M. Clark and C. Crawford (eds), *Legal Medicine in History* (Cambridge, 1994), 223–41. In 1852 when Dr John Campbell embarked on his career, he had expressed his doubts about separate confinement and its impact on the mind, commenting on the stupor, apathy and indifference of prisoners who had experienced it.^90^90J. Campbell, *Thirty Years Experience of a Medical Officer in the English Convict Service* (London, 1884), 105. After working in the prison system for 30 years, including a lengthy stint at Woking Invalid Prison, his views had changed, as he described how insane prisoners were often the ‘children of debased and drunken parents, generally of the habitual drunken class; so that the inherent hereditary predisposition, as well as the bad example set at home renders the removal from such baneful influences the surest safeguards’.^91^91*ibid*., 74–5. Cited in N. Davie, *Tracing the Criminal. The rise of scientific criminology in Britain, 1860–1918* (Oxford, 2005), 143. In 1868, the psychiatrist, Dr Thomas Laycock argued that the habitual/incorrigible criminal was essentially unreformable; a class of people so ‘constituted corporeally that they possess no self-control beyond that of an ordinary brute animal …. They are for the most part immoral imbeciles’.^92^92T. Laycock, ‘Suggestions for rendering medico-mental science available to the better administration of justice and the more effectual prevention of lunacy and crime’, *Journal of Mental Science*, 14 (Oct. 1868), 334–45, 334–5.

The changes in penal policy had a profound impact on local prisons. Evidence heard during the Kimberley Commission (1879), which, while primarily concerned with the operation of penal servitude in convict prisons, also heard details of harsh regimes in local prisons. A number of witnesses alleged that some inmates in local prisons languished in separation for as long as two years.^93^93BPP, *Report of the Commissioners appointed to Inquire into the Working of the Penal Servitude Acts*, Vol. I, 1879, (C.2368), 67, 135. At Liverpool, with its high rates of recidivism, there was considerable support for the explicitly penal approach of the new prison rules. In October 1864, Chaplain Carter had already called for the separate system to be made more rigorous, and he, along with Governor Jackson, claimed the new regulations would deter repeat offenders and alleviate overcrowding.^94^94LRO, 347 JUS/4/1/2, MJ, 27 Oct. 1864, Chaplain’s Report, 16. In 1863 and 1864 approximately 77% of Liverpool prisoners were serving sentences of one month or less; around 60%, and 70% of women, had been in custody prior to their committal to Liverpool Prison.^95^95*ibid.*, 9. Dr Archer also supported the new regime insisting its ‘enforcement’ would have ‘no perceptible ill effect upon the general health of the prisoners’.^96^96*ibid.*, 13. By 1867, Jackson claimed to have made ‘the prison as disagreeable to the prisoners as the Act of Parliament will allow and I have reason to believe the discipline is more strict than formerly’.^97^97*ibid*., 25 July 1867, Governor’s Report, 123.

The Liverpool justices, however, objected to the forms of labour – shot drill and stone breaking – recommended by Carnarvon’s 1863 committee.^98^98McConville, *English Local Prisons*, *op. cit*., 115–18. Liverpool’s justices were concerned that shot drill was ‘dangerous and likely to rupture prisoners’, while ‘stone breaking can only be enforced by constant punishment’.^99^99LRO, H365.32 BOR, Report of Visiting Justices of Borough Gaol (1865), 5–9. Both required close and expensive supervision ‘impossible in a large prison such as Liverpool’. Instead, they installed a treadmill and by 1868, it was in full operation with prisoners spending six to seven hours a day on it.^100^100LRO, 347 JUS/4/1/2, MJ, 29 Oct. 1868, Governor’s Report, 5. Once again, a confident Jackson claimed the ‘Treadmill … phase [of punishment] already proved a deterrent to the old thieves; and if it has not reformed them, it has had the effect of getting rid of a number who I have been informed have cleared out of Liverpool’.^101^101ibid.

Inspector Voules’ attempted to guard against the excessive enthusiasm for the stricter system at Liverpool, objecting to proposals that prisoners be denied exercise during the first 14 days of their sentence. Voules had observed the effects of the experiment at Wakefield Prison where similar practices had caused ‘injury’.^102^102LRO, 347 MAG/1/3/1A, Minute Book of the Visiting Justices Sessions Gaol and House of Correction, 19 Feb. 1856–25 Sep. 1866, 17 May 1864, 107. In his views on prison discipline, Voules demonstrated the enduring ambiguity among prison administrators. During his visits to local prisons, he observed harm being inflicted on prisoners by the system of discipline and the prison environment but retained his faith in the overall efficacy of the separate system. In his evidence to the 1863 Carnavon Committee, he noted that ‘unproductive employment’ such as the treadmill led to the degradation and irritation of prisoners’ minds and that separation was a ‘severe punishment’.^103^103Carnarvon Committee, 1863, Minutes of Evidence, 187, 192–3, 203. Yet he insisted that separation was ‘the only safe foundation of prison discipline … it forces a man to reflect; it makes him feel that employment is a boon … and it separates him from [the] contaminating influence of other prisoners’.^104^104*ibid*., 186.

In 1864, when advising the Liverpool justices on the new rules, Voules suggested the prison supply comfortable bedding and he opposed the practice of picking oakum in separate cells as it endangered prisoners’ health. Concerned about the potential impact the regime might have on prisoners’ minds, he advised they be given access to books of a secular nature and the chaplain granted discretionary power to allow prisoners with ‘great depression or inability to work’ to attend daily service in chapel.^105^105LRO, 347 MAG/1/3/1A, Minute Book of the Visiting Justices Sessions Gaol and House of Correction, 19 Feb. 1856–25 Sep. 1866, 17 May 1864, 106–8. The justices resisted some of his recommendations but most were accepted.^106^106*ibid*., 26 July 1864, 124.

The Liverpool justices recognized that the new regime was more taxing for prisoners and that some might not be physically or mentally able to withstand it. In July 1864, they instructed Governor Jackson to impress upon the doctors ‘the importance of watching the effects of these regulations with extra vigilance until they have been tested’, and discouraged the application of the full vigour of the rules to ‘prisoners whose constitutions are so much enfeebled by debauchery or disease to enable them to bear safely their full regime’.^107^107*ibid*., 118–19. While recognizing the increased burden on doctors in terms of their workload, the justices advised Jackson that

they regard this as the inevitable consequence of any proper system which would not be too indulgent for a robust prisoner, would be injuriously severe for one of delicate constitution or in bad health. It will also be needful for yourself and the other officers to watch the effects of these new Rules on the mental state of prisoners with a view to relaxing them in particular cases in which it may appear necessary on sanitary grounds.^108^108*ibid*.

The justices, though mindful of the potential risks the new regulations posed to the minds of prisoners, concluded these were inevitable consequences of a robust system of prison discipline.

Under the new rules, investigations into cases of insanity occupied substantial amounts of prison staff’s time. In 1864 only one prisoner, John Murray, a criminal lunatic, was sent to Rainhill, but a further two male prisoners were reported to have tried to hang themselves. Murray had been committed in October 1863 for six months and removed to Rainhill in January 1864 but escaped in April. He had been in Liverpool Prison several times and previously, while under sentence of penal servitude, was sent to Fisherton Asylum and was subsequently released. Of the two prisoners that attempted suicide, the governor doubted their intention to injure themselves. One was removed to Millbank Prison and the other, who was charged with desertion from the army, was escorted back to his regiment. During the year, six more prisoners, five male and one female, showed signs of insanity. The woman was ‘given up to her husband’ while the men were sent to Brownlow workhouse.^109^109LRO, 347 JUS/4/1/2, MJ, 27 Oct. 1864, Governor’s Report, 14. Staff enlisted the help of other prisoners when managing inmates who threatened or attempted suicide. In July 1867 ‘a male prisoner’ was found to have ‘opened a vein in his arm with a piece of glass and bled freely … He denied any intention of self-destruction’. The governor ‘thought it right to place two other prisoners with him for about three weeks’ to watch over him.^110^110*ibid.*, 25 July 1867, Governor’s Report, 125.

Prison doctors were constantly on the watch for malingerers, punishing those they suspected of dissembling. In December 1889 Thomas Bradley, who was serving 18 months for burglary and ‘already had 2 strokes with a birch rod’, was charged with ‘disturbing the prison by shouting’. The case was brought before the justices and the evidence of two wardens and the prison doctor, Dr Hammond, was heard. They confirmed that Bradley ‘was in his cell shouting and disturbing the prison’. Bradley claimed he ‘could not help it he could not keep his mouth still’ and that ‘he has put his handkerchief in his mouth to try and stop it’. Hammond swore ‘that the prisoner is of sound mind – I do not believe his excuses – he is fit to undergo the punishment … Bradley was ordered to have 18 lashes with the cat’.^111^111LRO, 347 MAG/1/3/3, Proceedings VC, 16 Dec. 1889, 140.

Liverpool’s visiting justices were aware that some prisoners were unable to withstand the full rigour of the treadmill due to the mental and physical toll it took on severely weakened bodies and minds; ‘many of the prisoners have led very wild lives, and bring with them the seeds of disease latent in their constitutions’.^112^112LRO, 347 MAG/1/2/2, Proceedings of the Justices of the Peace, 1 and 2 May 1872, 75. Among prisoners sentenced to hard labour, between 10 and 15% were found to be unfit for hard labour of first class due to poor bodily health.^113^113LRO, H365.32 BOR, Report VJ, 7. The severity was eased for some; the requirement that prisoners be exercised daily after the first two weeks of their sentences was revoked for those on the treadmill or crank.^114^114*ibid.*, 9. Nonetheless, the justices believed in the reformatory effects of the treadmill commenting that

the placing of prisoners for a time on the treadmill when they first enter the prison has also a very healthy influence over their subsequent prison careers. The fear of being put back on the treadmill if they misbehave or are lazy acts as a security for good behaviour and a stimulus to exertion.^115^115LRO, 347 JUS/4/1/2, MJ, 27 Aug. 1868, 173.

Captain J.R. Veitch, Jackson’s successor as governor, was also certain of its deterrent effects, claiming a reduction in the number of punishments among prisoners. Having ‘examined several prisoners who had been regularly at work on the treadwheel – most for fourteen weeks – with a view of ascertaining if the work … had injured their health in any way’, Veitch found that most did not think it affected health but they had lost ‘a little flesh’.^116^116LRO, H365.32 BOR, Reports of the Governor, Chaplain, Prison Minister and Surgeon of the Liverpool Borough Prison (Oct. 1869), Governor’s Report, 13. Archer, responsible for certifying prisoners as ‘fit’ for hard labour, agreed, concluding that ‘persons of a weak frame of body, and those encumbered with an unnecessary amount of fat, of course feel it the most’.^117^117LRO, 347 JUS/4/1/2, MJ, 29 Oct. 1868, Surgeon’s Report, 19. In his 1869 report, he observed it had a severe effect in only a few cases. While constant watchfulness on part of the prison officers, notably the doctors, was required, among the mass of prisoners, he argued, its effects were favourable. Nonetheless, one fifth of prisoners – 399 out of 2,023 – sentenced to hard labour on the treadmill were unfit and excused on medical grounds.^118^118LRO, H365.32 BOR, RGCMS, Surgeon’s Report, 26.

Chaplain Carter, while advocating a strict regime and seeking longer prison sentences, came to oppose the treadmill ‘due to its physical effects, which can result in irreparable injury and causes much anxiety’.^119^119*ibid*., Chaplain’s Report, 18. In 1870, he cooperated with William Tallack, Secretary of the Howard Association, on a newspaper campaign critiquing its use at Liverpool, published by Tallack in the *Liverpool Mercury*.^120^120*Liverpool Mercury*, 28 April 1870, 4. Increasingly, Carter clashed with fellow officers over the harshness of the Liverpool regime and the implications for prisoners’ minds. In April 1873, in a dispute with Governor Veitch, he contradicted Veitch’s claims that the number of recommittals of old offenders was on the decline, insisting they were ‘still in excess of what ought to be’.^121^121LRO, 347 MAG/1/2/2, Proceedings JP, Chaplain’s Report, 22 April 1873, 101. A year earlier, he had disagreed with prison doctor Dr E. Parker, who had been appointed as Archer’s replacement.^122^122LRO, 347 JUS/4/1/2, MJ, 29 Dec. 1869, 231. Parker complained that the chaplain interfered with his duties and they clashed on whether, under the 1865 Act, the chaplain retained the authority, granted by the 1855 rule, to intervene in matters of health. Cautioning Carter to be discrete, the justices reminded the men that it was the doctor’s duty to determine in ‘each case the question, often a very difficult one, of whether these penal conditions can be safely enforced’. The chaplain was to assist, in watching out for signs of mental disorders, implying he retained some expertise in identifying mental distress. They encouraged Carter to draw the ‘surgeon’s attention to … any prisoner whose health may appear to be suffering’ and advised against excessive interference as it ‘might harass without aiding surgeons’.^123^123LRO, 347 MAG/1/2/2, Proceedings JP, 1 and 2 May 1872, 75. Carter’s concerns about the severity of the Liverpool disciplinary regime persisted, however; seven months before his retirement in 1873, he openly questioned it, calling for a return to the older reformative system:

it may be worth while to … try the experiment now far milder and softer influences – addressing themselves more to the mind of the individual prisoners than coercive and harsh appliance directed against their physical constitutions may not be more successful.^124^124*ibid.*, Chaplain’s Report, 22 April 1873, 101.

## Unfit for a ‘vigorous’ regime

The regime at Liverpool did not halt the rise in prisoner numbers, which increased from 884 in 1866 to 1,011 for the second quarter of 1867 and to 20,478 in 1883, with most serving short sentences,^125^125LRO, 347 JUS/4/1/2, MJ, 25 July 1867, Governor’s Report, 123; 347 MAG/1/3/3, Proceedings VC, 3 March 1884, 40. resulting in continued overcrowding and difficulties enforcing separate confinement, especially on the female side. In October 1864 the governor reported that on the female side ‘every year except one (1861) [there were] more prisoners than could be provided for in separate confinement’ while on the male side for ‘four years out of nine … had a greater number than the number of cells’.^126^126LRO, 347 JUS/4/1/2, MJ, 27 Oct. 1864, Governor’s Report, 5. In 1869, 358 women had been thrown into the ‘very worst kind of association’, ‘frustrating the object for which the gaol was constructed on the separate system’.^127^127LRO, H365.32 BOR, RGCM, Chaplain’s Report, 14. During the 1870s there were attempts to ease the pressure by removing women to Lancaster Castle, though overcrowding persisted for the remainder of the century.^128^128LRO, 347 JUS/4/1/2, MJ, 29 Dec. 1869, 232; 347 MAG/1/2/2, Proceedings JP, 24 April 1873, 98; 25 July 1873, 107.

The number of prisoners maintained in local asylums also rose steadily, as did the cost to Liverpool Corporation, from £115 0s 5d in October 1869 to £211 17s 7d in October 1877.^129^129BPP, *31^st^ RIPGB*, 1866 (3715), XXXVII, 347; BPP, *35^th^ RIPGB*, 1871 (372), XXIX, 44, 59; LRO, JUS/4/1/2, MJ, 29 Oct. 1868, Surgeon’s Report, 19; H365.32 BOR, RGCM, Governor’s Report, 12; H352 COU, Borough of Liverpool, Proceedings of the Council, 1876–77, Reports of the Governor, Chaplain, Prison Minister, and Surgeon, of the Liverpool Borough Prison, 25 Oct. 1877, 545. In the year ending September 1877, nine prisoners were removed to the three Lancashire asylums of Rainhill, Prestwich and Whittingham.^130^130LRO, 347 MAG/1/2/2, Proceedings JP, 25 April 1872, 76. In most cases the prison doctors noted that the prisoners were insane on admission, disassociating the prison regime from responsibility for their mental collapse. Thomas Jones, a 29-year-old married labourer, was transferred in 1877. On admission to Rainhill, he was diagnosed with ‘mania and general paralysis’, declared ‘dangerous to others’, and ‘imagined the Queen was his grandmother’. He was ‘very stout’, ‘weak and shaky’, and had ‘old scars on his face and head and a bruise below his left eye’. His expression was described as ‘stupid’. There was a family history of insanity – both his father and brother had been in asylums. Jones had been in Liverpool Prison at least three other times for larceny. His mental state, already poor when he stood trial, steadily deteriorated and the morning after his admission to Rainhill he became ‘restless and mischievous’ and delusional. By May he was ‘dirty in his habits, very destructive of his clothing, constantly eating rubbish and daubing himself with excrement’. He eventually died in the asylum in November 1878.^131^131LRO, M614 RAI/11/6, Rainhill Asylum Male Casebook, Dec. 1873–July 1877, 331. The transfer of prisoners, both male and female, suffering from general paralysis of the insane from Liverpool Prison to Rainhill and other asylums was common in the latter part of the century, and it is likely that the prison was keen to be rid of such cases who were difficult to manage and who would eventually die of their disease. The rise in female cases, many removed from the prison, contributed to what was described as a remarkable rise in the disease in local asylums in the latter decades of the century.^132^132LRO, M614 RAI/8/6, Rainhill Asylum Female Casebook, Jan. 1870–Oct. 1873, 27; For more detail, see C. Cox, H. Marland and S. York, ‘Emaciated, exhausted and excited: the bodies and minds of the Irish in nineteenth-century Lancashire asylums’, *Journal of Social History*, 46 (2012), 500–24. These included Catherine O’Brien, a 30-year-old Irish woman imprisoned for stealing, who was described by her husband on her removal to Rainhill in April 1876 as addicted to drink. She died in Rainhill the following autumn. John Charles Smith, a 58-year-old labourer was admitted to Rainhill from Liverpool Prison in January 1896 and John Murphy, a 35-year-old married Irish labourer, was transferred in December of the same year. Smith died in Rainhill in September 1896, Murphy in January 1899.^133^133LRO, M614 RAI/8/7, Rainhill Asylum Female Casebook, Oct. 1873–July 1878, 194; M614 RAI/11/16, Rainhill Asylum Male Casebook, Dec. 1894–June 1896, 181; M614 RAI/11/17, Rainhill Asylum Male Casebook, June 1896–Nov. 1897, 105. For other examples of men transferred to Rainhill, many with general paralysis, see M614 RAI /11/2 Rainhill Asylum Male Casebook, Feb. 1857–May 1861, 113, 125, 130; M614 RAI/11/5 Rainhill Asylum Male Casebook, May 1870–Dec. 1873, 331; M614 RAI /11/16 Rainhill Asylum Male Casebook, Dec. 1894–June 1896, 144, 161, 169, 171, 181, 205, 215, 225; M614 RAI/11/17, Rainhill Asylum Male Casebook, June 1896–Nov. 1897, 95, 106, 125, 139, 172, 183.

Liverpool medical officers’ diagnosis of ‘real’ or ‘genuine’ insanity in these and other cases often followed prolonged periods of disruptive behaviour by prisoners involving the destruction of prison property, and very violent prisoners – and presumably those least welcome for that reason in the asylum – were most likely to be removed. While the comments of doctors recorded in the minutes of meetings are brief, they often refer to prisoners experiencing ‘delusions’ and to ‘excitability’, which prompted their destructive behaviour. Deliberations on the mental condition of prisoners – usually a dozen inmates on each occasion – took place at the regular meetings of the visiting committee, resulting in the certification of most cases to local asylums. A month after his admission to Rainhill John Murphy was reported to be ‘very noisy and violent and has marked grandiose delusions’, behaviour typical of cases of general paralysis, and a year later he remained ‘in a very restless and exalted state’.^134^134LRO, M614 RAI/11/17, Rainhill Asylum Male Casebook, June 1896–Nov. 1897, 105. Women also demonstrated volatile and destructive behaviour.^135^135David Nicolson, medical officer at Woking Prison, described these episodes as ‘breaking out’ though the term was not used at Liverpool: D. Nicolson, ‘The morbid psychology of criminals’, *Journal of Mental Science*, 19 (Oct. 1873), 398–409, 402. In July 1873 Frances Holden, a 33-year-old single woman, committed on numerous occasions to Liverpool Prison for prostitution, was transferred to Rainhill. She claimed she had been in prison 33 times and that ‘her child was an officer’ in Liverpool Prison. On admission to the asylum, she was described as suffering from mania and that she ‘at one time is very excited and at others more depressed’. She continued to engage in destructive behaviour in the asylum – throwing a basin through the window – and experienced delusions. In October 1876 she was removed ‘unimproved’ to Lancaster Asylum.^136^136LRO, M614 RAI/8/6 Rainhill Asylum Female Casebook, Jan. 1870–Oct. 1873, 278. For other examples see *ibid*., 129, 279; M614 RAI/8/18, Rainhill Asylum Female Casebook, Oct. 1895–July 1897, 16, 63, 73, 84, 85, 98, 206, 212, 250.

Large numbers of women continued to be admitted to Liverpool Prison, mainly on charges of prostitution, drunkenness or public order offences. In 1884, the Governor lamented the trouble this ‘incorrigible female class’ gave him.^137^137McConville, *English Local Prisons*, *op. cit*., 338. Of the 21,602 prisoners committed during the year, 9,084 were women who were reported to be troublesome and ‘not amenable to discipline’, more liable to reconviction than male prisoners, ‘mostly very ignorant’ and ‘indisposed to work in prison’.^138^138LRO, 347 MAG/1/3/3, Proceedings VC, Annual Report, 30 Jan. 1885, 46; BPP, *Report from the Departmental Committee on Prisons* [Gladstone Committee], 1895 (7702–1), LVI, Minutes of Evidence, 63. They were frequently punished for disrupting discipline – in 1884, 29 cases were brought before the visiting committee and 21 received punishment. The remainder were excused as ‘medically unfit’.^139^139LRO, 347 MAG/1/3/3, Proceedings VC, Annual Report, 30 Jan. 1885, 46. Their presence in prison, alongside the persistent problem of overcrowding ‘renders it impossible to carry out the solitary system and … matters of detail which render Gaol discipline effective and deterrent’.^140^140LRO, 347 MAG/1/2/2, Proceedings JP, Prison Minister’s Report, 28 July 1870,18. Many were also very ill. Annie Cochrane, an Irish prostitute, arrived delusional, intemperate and ‘dangerous to others’ in Rainhill in March 1906 after being removed from Liverpool Prison. She claimed that she had been in prison 30 times in the last 10 years for fighting and drunkenness, and that ‘she has never done any work, is not strong enough & has no heart for it’. In Rainhill her delusions of persecution continued and she repeatedly assaulted the charge nurse. Four years later she became quieter and died of phthisis in the asylum’s isolation wing in October 1910.^141^141LRO, M614 RAI/8/25, Rainhill Asylum Female Casebook, Feb. 1905–May 1906, 202.

Reverend James Nugent, Catholic minister to the gaol, was particularly disturbed by the high number of Catholic prostitutes, with almost two-thirds of the 1,526 prostitutes reported in 1864 being Catholic and most likely Irish.^142^142LRO, 347 JUS/4/1/2, MJ, 27 Oct. 1864, Prison Minister’s Report, 20–1. There were few opportunities for female migrant labour in Liverpool, and Nugent claimed ‘the destitute and friendless girl is readily allured into the path of crime’, the city of Liverpool becoming ‘the general refuge of the vicious and fallen’.^143^143ibid. To prevent women, many as young as 16, embarking on ‘the paths of defiant immorality’ and becoming repeat offenders, Nugent advocated for punishments that would act as a ‘warning to others as well as a means of reformation for themselves’.^144^144LRO, 347 MAG/1/2/2, Proceedings JP, Chaplain’s Report, 27 April 1870, 9. He also sought longer sentences for ‘drunkards’ who, he imagined, were a ‘heavier expense to the ratepayers than the Criminal Class’.^145^145*ibid.*, 28 July 1870, 18. While concluding that the ‘greater part of these poor creatures are thoroughly hardened in crime and humanly speaking utterly reclaimable’, he was hopeful for those ‘comparatively young in crime … in which great and lasting good may be done’.^146^146*ibid*., 45. Many, Nugent argued, were sincere in their efforts to ‘abandon vice’, and had been forced into the life through circumstance and not ‘wilful depravity or choice’. It was these helpless and hopeless cases, without food or shelter, who were forced through ‘bitter necessity to return to their old haunts and habits’, leading to high rates of reconvictions.^147^147*ibid*., Prison Minister’s Report, 27 April 1870, 9. Nugent and Carter called for various remedial measures and creative attempts were made at the prison to move the women away from Liverpool’s corrupting influence. For some, the cost of the passage back to Ireland was paid for, though several subsequently reappeared in Liverpool Prison.^148^148LRO, H365.32 BOR, RGCM, Chaplain’s Report, 22; 347 MAG/1/2/2, Proceedings JP, Chaplain’s Report, 28 July 1870, 45. A small number of women were discharged to the Catholic refuge and the Magdalen Asylum of the Good Shepherd,^149^149LRO, 347 MAG/1/2/2, Proceedings JP, Prison Minister’s Report, 28 July 1870, 22–3. while an active Discharged Prisoner’s Aid Society and a mission branch of the Church of England Temperance Society worked with the prison to provide financial support for female emigration.^150^150Gladstone Committee, 1895, Minutes of Evidence, 62; LRO, 347 MAG/1/2/2, Proceedings JP, Prison Minister’s Report, 27 April 1871, 44.

While contemporary medical explanations for the ‘incorrigibility’ of female prisoners increasingly suggested that their behaviour was the result of ‘some pathological condition rather than any ill will’, their continued presence provoked desperation among Liverpool Prison officials.^151^151Zedner, *op. cit*., 5. In the 1890s the Visiting Committee wrote to the Home Secretary seeking the introduction of a ‘system of reformatory detention’ for the women but they feared ‘there wd [*sic*] be difficulty agreeing to a proposal that Criminals – like lunatics – should be detained till they are cured’.^152^152TNA, HO 45/9695/A9757, Letter to the Home Secretary, 18 Feb. 1892. The sought for intervention was not forthcoming and the flow of female convictions to Liverpool Prison for prostitution, drunkenness and disorder continued unabated.

## Conclusion

Liverpool Prison both exemplified and exaggerated the problems faced by many local prisons, situated in a city that was experiencing rapid expansion, and characterized by high levels of migration, poverty, disease and crime. Many prisoners committed in the late Victorian period at Liverpool and elsewhere were physically weak and mentally distressed, without resources and likely to reoffend on leaving prison. They were quite simply the least appropriate prisoners to subject to the discipline of separate confinement and its many variations, for short or more prolonged periods. While some Liverpool Prison officers acknowledged the risks to prisoners’ minds and in some instances encouraged the amelioration of the prison regime, others were committed to its full implementation. However, the discipline, although adapted for local gaols in the 1850s and 1860s, was also imperfectly applied. In the overstretched and overcrowded environment of Liverpool Prison, this led to severe limitations in imposing separation, as did the constant throughput of short-term prisoners. In the final decades of the century, the large number of committals and the high incidence of short-term sentences and reoffending prompted support for the increasingly penal approach that was being imposed nationally after 1877, and attempts were made to apply separation as one aspect of a regime that increasingly emphasized deterrence. Under mentally and physically testing regimes and conditions in Liverpool, there was a heavy toll on prisoners’ minds. For some inmates, the disciplinary regimes of separate confinement and subsequent changes to penal policy exacerbated existing mental disorders while for others it provoked new incidences of mental distress.

Yet, while ambiguity persisted about prisoners’ ability to withstand separation and then increasingly penal regimes, prison officials were reluctant to mitigate them. They were slow to respond in many instances to cases of mental breakdown, suspicious of attempts to feign insanity, and retained mentally ill prisoners until they manifested destructive and violent behaviour. Implementing transfers to asylums, which would have eased the burden on prison doctors and other officers, was delayed – though less so in cases of general paralysis or where prisoners became particularly disruptive – not only because it led to increased costs for the local authorities but more significantly because it highlighted the failure of the prison to deter, reform and redeem.

Prisons were never intended to be places of medical treatment and cure; yet, while from the 1830s legislation endeavoured to divert mentally ill offenders away from prisons to asylums, mentally ill inmates accumulated in local prisons throughout the nineteenth century. This was especially remarkable at a time when advocates of specialist care for the insane in asylums, and the importance of early removals, were vocal and influential.^153^153See for example Andrew Scull, *The Most Solitary of Afflictions. Madness and society in Britain, 1700–1900* (New Haven & London), ch. 3, esp. 163–4. However, this article has demonstrated how prisons in effect became sites for the management of mental disorders. Most scholars of English prisons, including Henriques, Ignatieff and McConville, who have examined incidences of mental disorder in convict and local prisons, have been concerned with the implications for national penal policy. These included the modifications introduced to the model prison at Pentonville after its failure to introduce separate confinement in an extreme form in the 1840s and criticisms levelled against Edmund Du Cane during the Gladstone Inquiry of 1895, that highlighted the tendency of prison discipline to provoke mental disease and further debilitate weak-minded prisoners. By placing the management of mental health at the centre of our study of Liverpool Prison, this article has shifted the focus away from the development of penal policy to assess how attempts to enforce disciplinary regimes and environments affected prisoners’ minds and the practices of prison staff, especially the medical officers. Medical staff emerge as complex social actors whose responses to cases of mental disorder amongst their charges were ambiguous. They cannot be easily categorized as enforcers of penal policy bound by the constraints of dual loyalty, as argued by Sim, or, as Higgins and Hardy have suggested, doctors endeavouring to do their ‘professional best’. We have also revealed an under-researched outcome of the introduction of harsher penal policies in the late Victorian period. While much has been written about the failure of these policies to halt the growth of committals to English prisons and to deter habitual criminals, they also had a devastating impact on the mental well-being of inmates and their access to treatment. In Liverpool at least, these casualties appear to have been accepted as a price worth paying in the quest to punish and reform.

